# The Needs of Foster Children and How to Satisfy Them: A Systematic Review of the Literature

**DOI:** 10.1007/s10567-017-0246-1

**Published:** 2017-10-26

**Authors:** Anne Steenbakkers, Steffie Van Der Steen, Hans Grietens

**Affiliations:** Centre for Special Needs Education and Youth Care, University of Groningen, Grote Rozenstraat 38, 9712 TJ Groningen, The Netherlands

**Keywords:** Foster care, Foster families, Development, Needs, Need satisfaction, Systematic literature review

## Abstract

Family foster care deeply influences the needs of children and how these are satisfied. To increase our knowledge of foster children’s needs and how these are conceptualized, this paper presents a systematic literature review. Sixty-four empirical articles from six databases were reviewed and categorized (inter-rater agreement *K* = .78) into four categories: medical, belongingness, psychological and self-actualization needs. The results give a complete overview of needs that are specific to foster children, and what can be implemented to satisfy these needs. This study shows psychological needs are studied more often compared to the other categories, which specially relates to much attention for mental health problems. Furthermore, most articles focus on how to satisfy the needs of foster children and provide no definition or concrete conceptualization of needs. Strikingly, many articles focus on children’s problems instead of their needs, and some even use these terms interchangeably. This review illustrates that future research should employ a proper conceptualization of needs, which could also initiate a shift in thinking about needs instead of problems.

## Introduction

Worldwide, estimates are that 143 million children are separated from their birth families, and for most of these children (about 95%), family foster care is where they find a caring and nurturing home (Courtney et al. 2009; McCall 2011). Many foster children have a history of maltreatment and are struggling with behavioral problems and complex trauma (Greeson et al. 2011), which often cause placement disruptions (Eggertsen 2008). In addition, many of them experience out-of-home placement to be a great loss and feel lonely at the start of a foster family placement (Herrick and Piccus 2005; Schofield and Beek 2005). Foster parents are vital in providing a secure base for these children (Schofield and Beek 2005), enabling them to make a positive developmental turn and deal with their traumas (e.g., McLaughlin et al. 2012; Nelson et al. 2007). Meeting the needs of foster children provides them with a more stable and secure placement in which they can thrive (Berrick and Skivenes 2012). The needs of these children are therefore a recurrent theme in the literature.

### Basic Human Needs

The literature defines needs as necessities for a healthy development. Satisfying needs is a continuous process; successful need satisfaction leads to (further) growth and well-being, while failing to meet needs can inhibit this (Deci and Ryan 1985; Maslow 1943). Need satisfaction is formed by environmental factors, or changes in individual or interpersonal actions, thoughts or feelings (Deci and Ryan 2012; Maslow 1943).

Maslow (1943) was among the first to develop a theory encompassing both the biological and psychological needs of humans. According to this theory, people have physiological needs (e.g., the need for water and food), a need for safety, a need for love and belongingness, a need for self-esteem and a need for self-actualization. For any need, frustration results in increased desire, while satisfaction results in decreased desire, with the first needs mentioned being most desired when frustrated. Other need theories focus more on either survival needs, such as the terror management theory (Greenberg et al. 1997), or on psychological and self-actualization needs, such as the self-determination theory (Deci and Ryan 1985) and the core social motives theory (Fiske 2003).

 This article focuses on how needs are presented in the foster care literature. Maslow’s need hierarchy is used as theoretical framework, because of the broad range of needs it encompasses. That said, researchers have criticized this theory for emphasizing nature more than nurture, and for the inconclusive evidence of the hierarchical structure of needs (Neher 1991). Nonetheless, recent studies have successfully used Maslow’s hierarchy as a framework to examine children’s needs, such as for children in Kindergarten (Medcalf et al. 2013), children living in poverty (Noltemeyer et al. 2012) and children with disabilities (Lygnegård et al. 2013). This not only indicates the applicability of this theory to the needs of children today, but also for children growing up in specific and vulnerable conditions.

### The Needs of Foster Children

Children’s environment plays a significant role in defining the specific needs and how they can be satisfied (Deci and Ryan 2012; Harper and Stone 2003). Adverse experiences prior to care, the out-of-home placement and living in foster care cause children to develop specific needs (Berrick and Skivenes 2012). For example, children in foster care are at risk to develop medical, behavioral and emotional difficulties (Oswald et al. 2010; Smith et al. 2007), and their cognitive abilities and school achievements often lag behind (Jacobsen et al. 2013; Vacca 2008). In addition, foster children live apart from their biological parents. This disturbs the development of attachment and sense of belonging to their biological family, while they also have to form new relationships with their foster carers (Schofield and Beek 2005). Moreover, traumas experienced in their childhood can cause post-traumatic stress symptoms and internalizing behavioral problems (Greeson et al. 2011). Despite these circumstances, children in foster care are able to make a positive developmental turn when growing up in a secure and nurturing environment (McLaughlin et al. 2012; Schofield and Beek 2005). It is therefore important to satisfy children’s needs in an age-appropriate way, with their personal histories kept in mind (Berrick and Skivenes 2012). To our knowledge, however, there is no overview of the broad range of needs and how these can be satisfied specifically pertaining to children in foster care.

This article therefore focuses on two things: (1) systematically review the needs of children in care and the ways to satisfy them and (2) examine how the literature conceptualizes those needs. The aim is to create a coherent overview of the needs of foster children, useful for both researchers and practitioners. This overview can guide future research on the needs of foster children and assist practitioners when trying to meet the needs of foster children.

## Methods

A computer-based systematic literature search was conducted following the PRISMA statement (Moher et al. 2009). The search was conducted on June 14, 2017, using the databases ERIC, PsychInfo, Medline, PUBmed, Web of Science and Elsevier Science Direct. To identify articles related to foster children, the following search terms were included: (“foster child*” OR “child* in care” OR “child* in foster care” OR “foster care child*” OR “child* in substitute care” OR “substitute care child*” OR “child* in out-of-home care” OR “out-of-home care child*”), wherein ‘child’ was also substituted by ‘youth,’ ‘teen,’ ‘adolescent,’ ‘boy’ and ‘girl.’ Moreover, search terms were added that pertained to the needs of foster children: (need* OR demand* OR requir*).

The titles and abstracts of 2471 articles were read, and a selection of relevant articles was made on the basis of three criteria. First, although there were no constraints on publication date, only peer-reviewed empirical articles were included that were conducted in western countries. The empirical study should focus on cases of children, thus excluding policy analyses or studies inquiring other people (such as professionals and other stakeholders) about the needs of children in foster care as a group. Second, the main target group had to be children living in family foster care between the ages of 6 and 18. Younger children were excluded because self-actualization needs are less prominent for this age category. Articles covering a wide range of ages that also incorporated our target group of age 6–18 were included, but needs specific for younger children will not be described. We chose to focus on family foster care because this reduced the amount of variation between countries and welfare systems. Moreover, differences were expected in belongingness needs between children growing up in a family environment compared to group or residential facilities. Articles that compared children in family foster care to other groups of children were included, as well as studies on children in out-of-home care of which at least 70% of the target group consisted of children in family foster care. Third, the article had to focus on the needs of these children as directly stated in the title, abstract or keywords. This excluded articles that might pertain to the needs according to Maslow’s hierarchy, but do not name it as such. In addition, articles regarding the needs of care leavers and adolescent mothers in care were excluded. After this selection and deletion of duplicates, a total of 218 articles remained.

The full texts of the remaining articles were read by three researchers who again decided whether an article met the inclusion criteria. Most articles that were excluded in this phase did not describe the needs of children, but only mentioned the terms ‘needs’ once or twice without further explanation. Other reasons articles were excluded because they were conducted in non-western countries, did not adequately describe the sample of participants, were not empirical examinations of child cases or focused on the needs of foster parents. This final selection process resulted in 64 articles for this review.

While reading the articles, the researchers specifically searched for the term needs, requirements and demands in order to find the relevant information about these concepts. This information was used to summarize and discuss the reviewed articles in the results section. Additionally, the authors extracted from each article the definition of needs, target group (age, *N*, care setting), country of the study and the research methods employed to identify needs.

In order to cluster the needs, a categorization system was formulated based on the five needs of Maslow: physiological, safety, belongingness, self-esteem and self-actualization needs. When going through the articles, however, these categories were not sensitive enough to incorporate all articles and make a clear enough distinction between the various topics. Medical needs were often encountered within the articles, which pertains to both physiological needs (i.e., for food and water) and physical safety needs. No other needs regarding physiology were identified; thus, this category was named medical needs. The relational aspects of the safety needs were included in the belongingness category, which covered all aspects of relationships of children in foster care. The self-esteem needs, the need for prestige and accomplishment, were combined with other individual psychological needs, such as mental health, autonomy and coping. Therefore, this category was named psychological needs. Lastly, articles about education, leisure and employment were categorized within self-actualization needs. See Table 1 for a description of each need category.

**Table 1:** Overview of the four needs categories

Category	*N*	Description
Medical needs	21	Needs regarding physical health, physical development and treatment and identification of medical conditions
Belongingness needs	17	Needs regarding relationships with others, such as (foster) parents and peers, and related constructs, such as attachment and permanency
Psychological needs	43	Needs about (individual) psychological phenomena such as self-esteem, mental health, autonomy and coping
Self-actualization needs	14	Needs about learning, education, leisure and employment

Multiple categories per article are possible. *N*_total_ = 64

Two authors independently coded the final selection of articles according to these categories. An article had to be placed in at least one category, but could have as many as four category labels. The inter-rater agreement was calculated on the selected articles (90%, *p* < .0001, *K* = .78) and could be considered as good (Altman 1991), indicating similar coding and straightforward categories. Coding differences were subsequently discussed between the two researchers and resolved. As can be seen in Table 1, there was an uneven distribution of articles across the four need categories, with psychological needs being the most frequently mentioned. In the result section, the needs and how these can be satisfied will be described for each category, as well as common challenges mentioned in the literature. In line with human need theories, needs were considered as necessities for a healthy development, while satisfying needs can be accomplished by environmental factors and individual or interpersonal actions, thoughts or feelings that lead to a change in the level of need satisfaction (Deci and Ryan 1985; Maslow 1943). Furthermore, a section regarding challenges was added because many of the articles represented problems and other challenges as (an indication of) the needs of children in foster care.

## Results

As Table 2 depicts, the majority of the articles were written between 2000 and 2017 and conducted in Anglo-American countries. Other western countries are not or barely represented in the retrieved articles. Regarding the age of the children, 45% of the articles only included children within the age categories of 6–18, while 55% of the articles included a broad range of ages including younger children. To identify the needs of children, various methods such as standardized questionnaires (e.g., Child Behavior Checklist), case file analyses and interviews with people involved in foster care were employed. Most articles did not conceptualize or elaborate on the term needs (84%), but some provided an operational definition (e.g., scores on a questionnaire), defined children with high needs (those with physical handicaps or medical conditions) or provided a definition of specific needs (secure attachment).

**Table 2:** General characteristics of the articles in this review

*Year of publication*	
< 1990	1
1990–1999	6
2000–2009	21
2010–2017	36
*Country of conducted research*	
USA	39
Australia	8
UK	5
Canada	6
The Netherlands	3
Sweden	1
Ireland	1
Multiple (meta-analysis)	1
*Age range*	
6–12 years old	3
12–18 years old	12
6–18 years old	14
0–12 years old	5
0–18 years old	30
*Method of need identification* ^a^	
Standardized questionnaire(s)	23
Interview/survey foster children	20
Interview/survey professionals	11
Case files	18
Interview/survey foster parents	9
Child assessment	5
Open-ended questionnaire(s)	5
Other	2
*Definition of needs*	
No definition	52
Operational definition	10
Broad definition of high need children	1
Specific need defined	1

^a^Multiple categories per article are possible. *N*_total_ = 64

### Medical Needs

The medical needs of children in foster care are described in 21 articles and are commonly researched in combination with psychological needs (66%).

#### Needs

Although many articles indicate that children in foster care have more complex medical needs compared to their peers, the articles neglect to describe actual needs, but instead focus on medical problems and diseases. What can be concluded from the articles, however, is that children need to be physically and developmentally healthy, or at least as healthy as their specific medical conditions allow them to be. One study comments on this aspect, indicating that health screenings can only be effective when promoting health rather than screening for diseases (Hill and Watkins 2003).

#### Satisfying Needs

When medical problems are identified, personal treatment plans should be written, and treatment and other services should be implemented in order to improve the health outcomes of children in foster care (Rodrigues 2004; Rubin et al. 2004). What these services are, differs per health problem, but a multidisciplinary team should preferably determine the treatment plan (Kaltner and Rissel 2011). Adolescents specifically need tailored interventions to stimulate safe sex and to prevent early pregnancy and STD’s (Becker and Barth 2000). A study among 442 foster parents showed that 83% of the foster parents are convinced that the medical needs of their foster child are met (Hayes et al. 2015). Foster parents caring for children with complex medical conditions indicate that training helped them provide the necessary care for these children (Lauver 2008).

#### Challenges

Many articles report on the increased medical health problems of children in foster care; depicting medical problems as medical needs. Studies differ with regard to the reported prevalence of medical problems, ranging from one-third of the foster children (Ringeisen et al. 2008; Sullivan and van Zyl 2008), to about half of them (Steele and Buchi 2008; Takayama et al. 1998) and even up to 90% (Chernoff et al. 1994; Hochstadt et al. 1987; Nathanson and Tzioumi 2007). Based on over 30.000 case files, about 6% of children in foster care have so-called complex needs, which means having co-occurring physical health problems, emotional problems and the need for specialized services (Yampolskaya et al. 2014). The most common health problems mentioned in the literature are incomplete immunization (Hill and Watkins 2003; Kaltner and Rissel 2011; Kling et al. 2016; Nathanson and Tzioumi 2007; Raman and Sahu 2014; Rodrigues 2004), vision problems (Chernoff et al. 1994; Nathanson and Tzioumi 2007; Steele and Buchi 2008; Takayama et al. 1998), and respiratory problems (Nathanson and Tzioumi 2007; Ringeisen et al. 2008; Rodrigues 2004; Takayama et al. 1998). Other medical conditions that are also often encountered in the foster care population are obesity, dental problems, skin conditions, STD’s, infections and allergies. A complete list is beyond the scope of this article, so we refer the reader to the above articles and these additional manuscripts (Arora et al. 2014; Becker and Barth 2000; Lauver 2008; Ogg et al. 2015; Rubin et al. 2004). While many articles report children in foster care have higher rates of medical health problems (e.g., Ringeisen et al. 2008), a study by Raman and Sahu (2014) did not find any differences between children in foster care and children at risk living in with their parents. The identified risk factors for developing medical problems are being male, being older, having a longer stay in foster care and having had multiple placements (Ringeisen et al. 2008; Rubin et al. 2004; Sullivan and van Zyl 2008).

Children should be assessed and screened for medical conditions by a multidisciplinary team of health professionals (Kaltner and Rissel 2011; Ogg et al. 2015; Rodrigues 2004), which should be administered as soon as a child comes into foster care (Chernoff et al. 1994; Steele and Buchi 2008). Nevertheless, not all children receive a medical examination (Rodrigues 2004). Nathanson et al. (2009) argue that screening is not only important when entering care, but also throughout the foster care period. Lastly, many studies have identified a major gap between the medical issues of children in foster care and the services provided (Feigelman et al. 1995; Hill and Watkins 2003; Kaltner and Rissel 2011).

### Belongingness Needs

Seventeen articles on belongingness needs of children in foster care were found. While most articles focus on (foster) family relationships, other adults and peers are also mentioned.

#### Needs

Children in foster care generally need continuity of the relationships with their birth family members (Kufeldt et al. 1995; Mason 2008). Especially sibling contact can be a point of continuity in unstable times, as they have lived in the same circumstances and had similar experiences (Kothari et al. 2014; Waid and Wojciak 2017). Establishing caring and supportive relationships with the foster family is considered a crucial need of children (Bell et al. 2015; Kufeldt et al. 1995; Mason 2008; Quest et al. 2012). Preferably, these relationships are characterized by secure attachments, a sense of permanency, mutual trust and emotional intimacy (Ashley and Brown 2015; Steenbakkers et al. 2016; Mason 2008). Schofield and Beek (2009, 2005) illustrate how infant attachment concepts also apply to children and adolescents in family foster care, because they need their foster parents to provide a secure base. Besides (foster) family members, other adults, such as a neighbor or family friend, and professionals can play an important role in the social networks of children in foster care. These people can provide emotional and practical support, and a sense of stability and continuity of relationships (Bell et al. 2015; Clausen et al. 2012). Lastly, friends and positive peer interactions are an important need of children in care (Mason 2008).

#### Satisfying Needs

In order to establish loving relationships with foster parents, children should be provided with a stable, affectionate and safe home environment (Fernandez 2008; Kufeldt et al. 1995). Foster parents can create a secure base for children in their care by being available, helping them manage their behavior and feelings, building their self-esteem, helping them feel effective and helping them to belong in the foster family (Schofield and Beek 2005, 2009). A high perceived quality of caregiver relationship can lower the risk of depression for children in foster care (Guibord et al. 2011). Children indicate that at the start of a placement foster parents can help them by showing an understanding of the difficulties of coming into care, and help them to become familiar with their new home, routines and responsibilities (Mitchell et al. 2010). Conversations with foster parents about their past, when characterized by trust and interest, can contribute to youth finding emotional support from their foster parents (Steenbakkers et al. 2016). A culturally sensitive facilitator to meet the attachment needs of African-American youth is by assisting them with their hair care (e.g., braiding), since this provides the opportunity for healthy touching and nurturing (Ashley and Brown 2015).

Contact with birth family members can repair disrupted ties, and children with more contact tend to view their parents more positively (Kufeldt et al. 1995). To facilitate sibling contact, specific interventions have been established that promote sibling contact and support (Kothari et al. 2014; Waid and Wojciak 2017). Lastly, children in foster care sometimes require help to understand and manage the complex family relationships with their birth and foster family (Kufeldt et al. 1995; Quest et al. 2012).

Supportive relationships with other adults should be characterized by a sense of safety, positive regard and commitment (Clausen et al. 2012; Fernandez 2008; Mason 2008; Quest et al. 2012). Moreover, these relationships with adults can help youth to learn social skills (Clausen et al. 2012), as well as give them tools to take on future obstacles (Guibord et al. 2011).

#### Challenges

Children in foster care can have difficulties with establishing and maintaining social relationships. Foster children show less prosocial behavior (Fernandez 2008), and a recent meta-analysis shows that age-appropriate social functioning does not improve during foster family placement (Goemans et al. 2015). Compared to other types of out-of-home care, children in family foster care require specific attention for attachment-related difficulties (Leloux-Opmeer et al. 2017). Contact with their birth family can be problematic due to the problems of their birth parents (Kufeldt et al. 1995). Lastly, children are at risk to experience abuse while in foster care by their foster parents, birth parents or other children. Steps should therefore be taken to protect children, especially those who already experienced abuse prior to care (Hobbs et al. 1999).

### Psychological Needs

In total, 43 articles explicitly focus on the psychological needs of children in foster care, which is about two-thirds of the articles selected for this review.

#### Needs

The well-being and everyday functioning of children in care depends partly on them developing self-esteem (Coholic et al. 2009; Fernandez 2008). LGBTQ youth in foster care specifically need to develop a positive self-identity about their sexual orientation (Gallegos et al. 2011). Children in foster care were often exposed to multiple traumas at a young age, and therefore, they need to learn how to cope with past experiences and construct a coherent life story (Coholic et al. 2009; Nathanson and Tzioumi 2007; Steenbakkers et al. 2016). Similar to the medical needs category, articles about mental health needs focus on mental illness and problems, with the exception of one article (Hill and Watkins 2003).

#### Satisfying Needs

Attentive and sensitive parenting is important for satisfying the needs for self-esteem, coping skills and self-regulation skills (Fernandez 2008; Gallegos et al. 2011; Mitchell et al. 2010; Schofield and Beek 2005, 2009; Stoner et al. 2015). Specifically, children in foster care need the people around them to understand their personal history; so that their environment can be sensitive to the signals they convey (Steenbakkers et al. 2016). This is important because a better adjustment to trauma has been indicated as a good predictor for reduction in depression (Stoner et al. 2015). In order to satisfy the need for autonomy and individuality, children should be included in the decisions about their care (Mason 2008).

When mental health problems are present, children should preferably receive individualized treatment and care in order to meet their mental health needs (Cantos and Gries 2010; Ringeisen et al. 2008; Rodrigues 2004; Shin 2005; Steele and Buchi 2008; Sullivan and van Zyl 2008; Takayama et al. 1998). Studies show that mental health service use by foster children ranges from 25 to 53% (Bellamy et al. 2010; Petrenko et al. 2011; Rodrigues 2004). When treatment is provided, it is important to differentiate among children based on their characteristics and maltreatment history (Bell et al. 2015; Reifsteck 2005). In addition, the required facilities should be close to the foster home and continuously accessible (Arora et al. 2014). Regarding the type of treatment, authors have argued that mental health needs are often treated with multiple psychotropic medications, while the child might be better off with therapeutic interventions (Coholic et al. 2009; McMillen et al. 2004), wherein the relationship with the therapist is key (Clausen et al. 2012). Moreover, adolescents are likely to receive the most invasive and stigmatizing mental health services such as inpatient and residential programs, while not always receiving community based services before this (McMillen et al. 2004). Some authors suggest to not only treat the child as client, but also the family and community around the child (Love et al. 2008; Yampolskaya et al. 2014). In addition to providing help, youth themselves also seek out help for their mental health issues, which is affected by their expectations of the care system and previous help-seeking experiences (Johnson and Menna 2017).

#### Challenges

The articles about mental health needs additionally provide information about the prevalence and types of mental health problems, often named in articles as mental health needs. Although the operationalization differs between studies, prevalence of mental health problems among foster children seems to fall between 44 and 66% (Arora et al. 2014; Bellamy et al. 2010; Maaskant et al. 2014; McNicholas et al. 2011; Scozzaro and Janikowski 2014). While this prevalence is high, children in family foster care have fewer mental health issues compared to children living in more restrictive out-of-home placements (Lardner 2015; Leloux-Opmeer et al. 2017; McNicholas et al. 2011). Around 6% of children in foster care seem to experience a complex combination of medical and mental health problems (Yampolskaya et al. 2014). The mental health issues mentioned are related to dealing with separation and loss (Chernoff et al. 1994; Nathanson and Tzioumi 2007), exposure to drugs and alcohol (Chernoff et al. 1994), emotion or behavior regulation (Arora et al. 2014; Bell et al. 2015; Bellamy et al. 2010; Fernandez 2008; Goemans et al. 2015; Guibord et al. 2011; McMillen et al. 2004; McNicholas et al. 2011; Ogg et al. 2015; Ringeisen et al. 2008; Rodrigues 2004; Steele and Buchi 2008; Stoner et al. 2015; Sullivan and van Zyl 2008; Yampolskaya et al. 2014), sexual abuse or inappropriate sexual behavior (Chernoff et al. 1994; McMillen et al. 2004), self-harm and violent behavior (Chernoff et al. 1994; McNicholas et al. 2011) and substance abuse (Gabrielli et al. 2016; Guibord et al. 2011). Mental health problems are positively correlated with the child’s current age, the age at the time of placement, maltreatment history and the number of placements (Cantos et al. 1996; Gabrielli et al. 2016; Maaskant et al. 2014; Shin 2005; Steele and Buchi 2008).

Mental health problems of children in foster care should be regularly screened and assessed in order to provide timely interventions, preferably by multiple informants (Cantos and Gries 2010; Nathanson et al. 2009). Although many foster children receive mental health services, some authors warn about a gap between foster children’s mental health issues and the referral rate (Fontanella et al. 2015; Hill and Watkins 2003; Kaltner and Rissel 2011; Ogg et al. 2015; Petrenko et al. 2011; Shin 2005). Only 23% of foster parents are assured that the mental health needs of their child are met (Hayes et al. 2015). A meta-analysis by Goemans et al. (2015) shows that internalizing and externalizing behavioral problems do not improve during placement in a foster family, questioning the effectiveness of treatment and care children receive. That said, a recent study reports adequate service delivery to 128 children in foster care with mental health problems in the USA (Scozzaro and Janikowski 2014).

Two additional issues are mentioned regarding the psychological needs of children in foster care. First, the multiple traumas children were exposed to during their youth can have a negative impact on their psychological development (Leloux-Opmeer et al. 2017). Secondly, overprotection and forced support can have a disempowering effect on children and does not meet their need for autonomy (Mason 2008).

### Self-Actualization

The literature on children’s self-actualization needs is very recent, with 12 out of 14 articles (86%) written in the last decade.

#### Needs

While all articles in this need category focus on the educational outcomes of children in foster care, most study how this can be accomplished (need satisfaction) and what hinders children to achieve well in school (challenges). The majority of the articles focus on education, except one article that showed that participation in extracurricular activities lowers the risk of substance abuse and depression among children in foster care (Guibord et al. 2011).

#### Satisfying Needs

Stability and connection to the same school can greatly assist children with completing their education (Piescher et al. 2014). Foster parents should support children with their school career and provide stimulation and input for their cognitive development (Fernandez 2008; Mendis et al. 2015; Zetlin et al. 2010). In addition to foster parents, other significant adults can stimulate youth to go to school and help with decisions about school, work and college (Hudson 2013; Mendis et al. 2015; Quest et al. 2012). When children are experiencing learning difficulties, a range of targeted interventions can be implemented. Although a study looking into the benefits of a home-based tutoring program was unable to find significant improvements (Zinn and Courtney 2014), other research suggests the benefits of services such as remedial teaching, additional classroom assistance, speech and reading interventions, and an educational support program (Petrenko et al. 2011; Tyre 2012; Zetlin et al. 2010). Youth themselves indicate a myriad of approaches to meeting their educational needs, indicating that there is not a ‘one-size-fits-all’ solution (Mendis et al. 2015).

#### Challenges

The literature often reports on educational difficulties, specifically in relation to learning difficulties (Leloux-Opmeer et al. 2017; McNicholas et al. 2011), and special education (Geenen and Powers 2006; Zetlin et al. 2010). Children in foster care seem to lag behind on cognitive measures, such as math and reading (Piescher et al. 2014), or have mental health or behavioral problems that interfere with learning (Zetlin et al. 2006, 2010; Zinn and Courtney 2014). Children with disabilities are argued to be at greater risk to have their educational needs overlooked (Geenen and Powers 2006), while children in family foster care have lower risks of having unmet educational needs compared to youth placed in residential facilities (Leloux-Opmeer et al. 2017). Authors also comment on the difficulties children in foster care encounter to receive educational services, such as delays in service provision after a school change (Petrenko et al. 2011; Zetlin et al. 2010). This calls for better communication between schools and welfare services to overcome cross-system barriers (Geenen and Powers 2006; Petrenko et al. 2011; Piescher et al. 2014). Lastly, researchers indicate the importance of comprehensive developmental and educational screening to identify children’s special educational needs (Petrenko et al. 2011).

## Discussion

The reviewed articles provide a varied picture of the needs of children in foster care, divided into four categories based on Maslow’s theory and adapted to the specific needs of foster children as depicted in the international literature. These four categories give an up-to-date overview of the specific needs children in foster care can experience and how these can be satisfied. Contrary to Maslow’s theory, the foster care literature does not focus on self-esteem needs, but on psychological needs in a broad sense, such as mental health, coping and identity development. Foster parents are often mentioned with regard to satisfying the needs of the children in their care, highlighting the importance of foster parent selection, training and support. Needs in a certain category that are met can positively influence the satisfaction of other needs. For example, sensitive caregiving from foster parents not only satisfies children’s need to belong, but also their psychological needs and learning opportunities, and prevents mental health problems. Likewise, unmet needs in one category limit opportunities to satisfy needs in other categories, such as mental health problems that interfere with learning. Whether these influences follow Maslow’s proposed hierarchy (in the sense that higher-order needs can only be satisfied when lower order needs are met) cannot be deduced from the reviewed articles and requires further analysis.

Regarding the conceptualization of needs, it seems that only a few studies describe needs in a way that aligns with Maslow’s original definition. More often, studies focus on how needs can be satisfied and what challenges children in foster care face to have their needs met. This could be explained by the fact that Maslow’s hierarchy of needs is universal; hence, the core needs of children in foster care are of little interest in the international literature, because they may be similar to those of other children. What seems to differentiate children in foster care from other children are the ways their needs are satisfied, and the high amount of challenges they encounter.

Strikingly, many studies focus on the *problems* of children and use the terms needs and problems interchangeably. This can be explained by the high impact of problem behavior on foster parents and the increased chance of breakdowns (Eggertsen 2008), and the use of instruments that measure current problems. Theoretically, however, these terms are not synonymous, given that meeting needs promotes a healthy development, while problems impede this (Maslow 1943). Problems can only be indicative of severely frustrated needs for which external satisfaction should most urgently be implemented. As argued by Hill and Watkins (2003), screening for problems can only be effective when it promotes need satisfaction. However, the absence of problems is not an indication of satisfied needs. In order to avoid undue confusion, a proper conceptualization of needs is necessary that differentiates the needs of children in foster care and the ways to satisfy them from the problems they encounter. Research can benefit from applying a holistic approach to children’s needs, by presenting them as necessities for a healthy personal development, and by incorporating both unmet and satisfied needs. This holistic approach can initiate a shift from thinking about problems to thinking about what can be done to meet certain needs.

### Strengths and Limitations

A strength of this study is that the analyses were based on a theoretical framework, yet adapted to the specific population of foster children. Besides presenting an overview of these needs, divided into four categories, we also separately reported needs and how to meet these. This distinction offers additional theoretical insight for researchers and gives practitioners the possibility to match children’s needs with actions and treatments suitable to meet these needs.

This study’s categorization of the literature was based on Maslow’s theory (1943), because of its broad range of needs specified. However, as indicated in the introduction, this theory has sound critiques, in the sense that it emphasizes nature more than nurture, and the insufficient empirical evidence for the hierarchical structure of needs (Neher 1991). Furthermore, the use of the word ‘need*’ in our search terms might have prevented us from including articles that describe necessities for a healthy personal development, but are not naming them as needs. Although we tried to mitigate this by also incorporating the terms ‘requir*’ and ‘demand*,’ other related constructs such as well-being, resilience and protective factors were not included in this review. Furthermore, we did not specifically search for terms directly related to the need theories, such as belongingness, self-esteem, self-actualization and self-determination. Although we assume that these constructs would be accompanied by the term ‘need,’ we acknowledge that this assumption might not apply to all papers.

Our study spanned several western countries, but many countries are not represented in the retrieved articles. Moreover, the countries that were included have different child welfare systems, for instance related to permanency planning for children placed out-of-home, and different conceptualizations of foster care, for example the inclusion or exclusion of residential facilities and the focus on kinship or non-kinship care (Gilbert et al. 2011). Although the influence of these differences on the needs of children might be limited, it could impact the preferred ways to satisfy needs, since countries have their own laws, policies and preferred interventions when assisting foster families.

Finally, our inclusion criteria allowed for a broad inclusion of studies, which increases the risk of bias in the selected studies. At a minimum, studies had to adequately describe their participant sample and how needs were obtained to meet our inclusion criteria, but we did not differentiate between random samples and convenience samples, the validity of the measures used or other potential biases (Viswanathan et al. 2013).

### Future Directions

In addition to providing a proper conceptualization of needs and initiating a shift in thinking about needs instead of problems, this review reveals three other key points for practice and future research. First, more research is necessary about children’s physiological needs besides medical health, psychological needs besides mental health and self-actualization needs besides education. While screening at the start of a placement ensures foster families can satisfy children’s basic physiological needs (such as enough food and clothes), the question remains if this is sufficiently monitored throughout a placement. In addition, although mental health problems of children are a great concern for their caretakers, other psychological needs such as identity and autonomy development should be more often researched in order to satisfy these needs. Moreover, while the need to receive an education is important for children, self-actualization includes more than just education, and can be achieved through leisure and hobbies.

Secondly, most articles do not mention children’s own possibilities of meeting their needs (e.g., seeking distraction, using coping skills). This gap devalues the agency and capabilities of children and might limit foster carers and professionals to explore the possibility of youth aiding in satisfying their own needs.

Finally, many studies use instruments that result in a measure of problems (e.g., problem screening questionnaires). Although interviews with experts, foster parents and foster children are employed to bridge this gap, these can be time-consuming to use in larger samples. A questionnaire could therefore be developed that validly determines the needs and the level of need satisfaction of children in foster care. Such an instrument not only enables researchers to make more generalized and valid statements about the needs of children in foster care, but could also be utilized in practice as an assessment and monitoring tool.
